# Embracing interactions in ocean acidification research: confronting multiple stressor scenarios and context dependence

**DOI:** 10.1098/rsbl.2016.0802

**Published:** 2017-03-29

**Authors:** Kristy J. Kroeker, Rebecca L. Kordas, Christopher D. G. Harley

**Affiliations:** 1 Ecology and Evolutionary Biology, University of California Santa Cruz, Santa Cruz, CA 95060, USA; 2 Life Sciences, Imperial College London, London SL5 7PY, UK; 3 Zoology and Institute for the Oceans and Fisheries, University of British Columbia, British Columbia, Canada V6T 1Z4

**Keywords:** cumulative impacts, climate change, ocean acidification, thresholds

## Abstract

Changes in the Earth's environment are now sufficiently complex that our ability to forecast the emergent ecological consequences of ocean acidification (OA) is limited. Such projections are challenging because the effects of OA may be enhanced, reduced or even reversed by other environmental stressors or interactions among species. Despite an increasing emphasis on multifactor and multispecies studies in global change biology, our ability to forecast outcomes at higher levels of organization remains low. Much of our failure lies in a poor mechanistic understanding of nonlinear responses, a lack of specificity regarding the levels of organization at which interactions can arise, and an incomplete appreciation for linkages across these levels. To move forward, we need to fully embrace interactions. Mechanistic studies on physiological processes and individual performance in response to OA must be complemented by work on population and community dynamics. We must also increase our understanding of how linkages and feedback among multiple environmental stressors and levels of organization can generate nonlinear responses to OA. This will not be a simple undertaking, but advances are of the utmost importance as we attempt to mitigate the effects of ongoing global change.

## 1. Introduction

Environmental change, which encompasses a wide range of physical and chemical changes, is outpacing our ability to forecast its consequences. Several issues limit our understanding of the emergent effects of these changes. First, CO_2_-driven environmental change comprises a suite of stressors with different and sometimes opposing patterns of occurrence and effects on species. Interactions between OA and other environmental stressors, defined here as natural or anthropogenic pressures that cause measureable biological responses, both positive or negative [1], can determine species responses [2,3]. Context is critical for forecasting the ecological effects of OA, and studies spanning a wide range of conditions are crucial to accurately interpret experiments. Second, the combined effects of multiple stressors on individual species will be mediated by the interactions with other species in an ecosystem [4]. Thus, studies are needed in diverse, functioning ecosystems that incorporate species interactions and compensatory dynamics.

**Figure 1.**
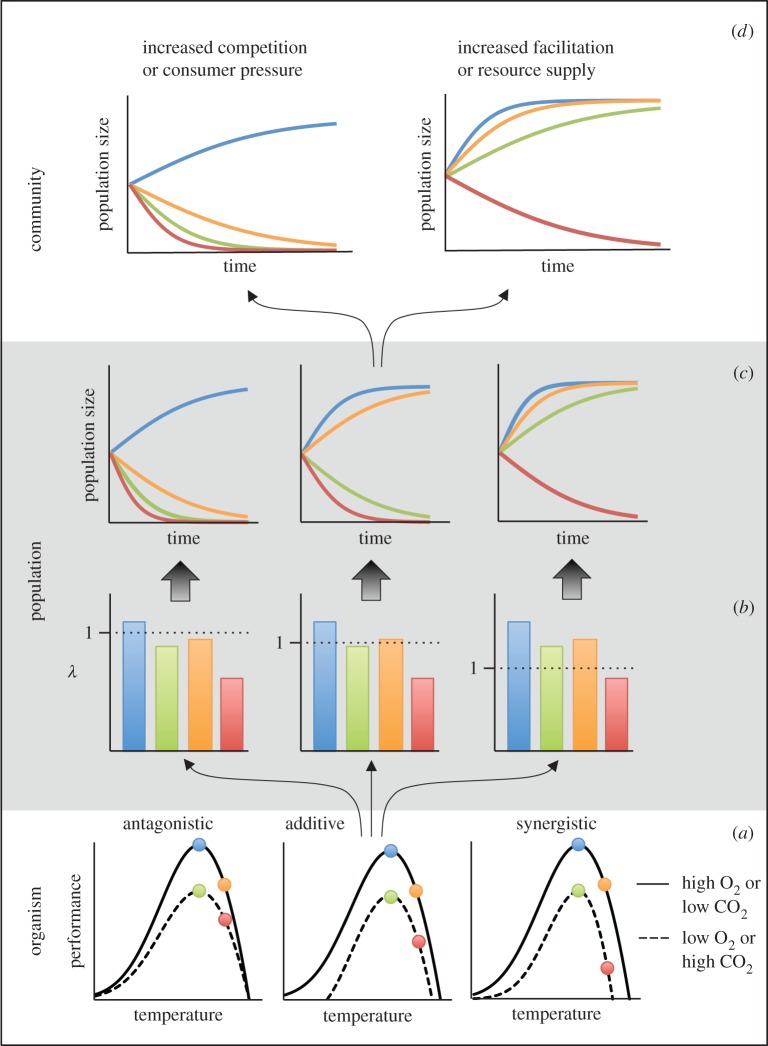
Conceptual figure highlighting how non-additive effects of environmental change drivers can arise within an organism, population or community despite a lack of non-additivity at lower levels of organization. For a single species exposed to two drivers (e.g. warming × high CO_2_ or low O_2_), the coloured symbols represent (*a*) changes in individual-level performance (circles), (*b*) intraspecific population responses at a static point in time (bars), (*c*) intraspecific population growth trajectories (lines) based on the scenarios presented in panel (*b*), and (*d*) alterations to one such set of growth trajectories (panel *c*, centre) when influenced by negative (left) or positive species interactions (right). For all graphs, blue represents the current ‘control’ conditions, green represents acidification (or low oxygen), orange represents warming, and red represents the simultaneous application of both stressors. The dotted line in panel (*b*) represents zero population growth (*λ* = 1). In these examples, a change in thermal performance with exposure to low oxygen or high CO_2_ can create antagonistic, additive or synergistic effects [12] (*a*). Species physiological responses can result in population persistence or extinction if growth rates are pushed past a demographic threshold (*b,c*). Interactions among species can also push populations past demographic thresholds (*d*), as when negative species interactions reduce population growth rates (shifting the orange trajectory from growth (in panel (*c*), centre) to decline (in panel (*d*), left)) or when positive interactions enhance population growth rates (shifting the green trajectory from decline (in panel (*c*), centre) to growth (in panel (*d*), right)). In such cases, indirect effects can override the direct effects at lower levels of organization.

Interactions among multiple environmental stressors, where the ecological effect of one is dependent on the magnitude of another, are very common across ecosystems [5–7]. These interactions can lead to non-additive outcomes, where the combined effects are more or less than expected (synergistic or antagonistic, respectively) compared with an additive or multiplicative model. However, our ability to predict interaction outcomes is very limited [6,8]. Several recent reviews highlight the need for a more mechanistic understanding of the physiological responses to OA and multiple stressors [9] and suggest a framework for scaling up these physiological effects to ecosystems [10,11]. Here, we expand that perspective to discuss how the underlying causes of non-additive outcomes of OA and other stressors may be due to interactions at or among several levels of organization (figure 1).

## 2. Interactions within the environmental milieu

Increasing atmospheric CO_2_ concentrations are causing a wide range of physical and chemical changes both on land and sea that interact [13]. For example, temperature influences seawater carbonate chemistry, such that warming will affect OA by decreasing CO_2_ solubility and affecting the dissociation coefficients of the carbonate system, leading to higher saturation states given the same CO_2_ concentration [14]. Environmental change is also likely to affect other physical and chemical factors associated with organismal performance. For example, the biological availability of heavy metals in the environment is enhanced by a reduction in pH, which increases the toxicity of these contaminants [12]. In cases such as these, researchers must ensure that OA effects are examined at appropriate levels of potentially interacting factors [15] and recognize that the ecological effects of OA may vary spatially and temporally, resulting in a mosaic of effects due to overlapping and interacting factors [3].

## 3. Interactions within an organism

At the physiological level, changes in an environmental stressor may exert selective pressure on traits that increase susceptibility or tolerance to a second stressor, such as OA [16]. The combined effect is in part due to whether both stressors stimulate or impair similar or different physiological pathways. For example, a stressor can increase susceptibility if it impairs a pathway that is critical in mounting a response to a second stressor (e.g. low oxygen levels preventing organisms from mounting a heat shock response to warming [17]). By contrast, a stressor can increase tolerance if it activates a pathway that is used in response to a second stressor [18]. For example, exposure to elevated temperature can prepare an organism to elicit a stress response to low oxygen [19]. In all of these scenarios, the history of exposure to the different stressors can further define the outcomes [9].

One simplified way to envision nonlinear outcomes of multiple stressors is to consider each stressor as having a threshold beyond which performance is inhibited, but the position of this threshold is dependent on the level of additional, interacting factors [20], such as OA lowering species' upper thermal lethal limits [21] (figure 1*a*). Elucidating the energy budgets for species of concern may provide a framework for incorporating the effects of interactions among multiple stressors. For example, a non-additive outcome of exposure to multiple stressors may arise when energy expended via maintenance metabolism exceeds energy gained through photosynthesis or consumption, creating a tipping point beyond which exposure results in death [20].

## 4. Threshold dynamics in populations

At the population level, non-additive outcomes could arise through the additive accumulation of effects on physiological processes when the cumulative effect crosses a demographic tipping point. For example, even a small, additive effect of a second stressor could cause population growth rate to switch from positive to negative (figure 1*b*). If sustained, the consequences of such combined effects may result in local extinction when the drivers co-occur (figure 1*c*). Thus, additivity at one level of organization does not preclude non-additivity at another.

## 5. Interactions within a community

Environmentally mediated changes in *per capita* interaction strength of species with strong influence on the community, including keystone species [22] or ecosystem engineers [23], can also have cascading effects on the abundance of other species. Even small increases in the abundance or *per capita* effects of competitors and consumers that nudge population growth rates of a focal species downwards could cause non-additive outcomes in response to OA if the population is pushed past key demographic thresholds (figure 1*d*, left). By contrast, increased resource availability (and the concomitant reduction in competition) or increased facilitation via habitat provision can increase population growth rates, potentially pushing the population past the threshold from negative to positive growth (figure 1*d*, right). Thus, non-additive effects of multiple stressors can arise in populations in a community setting due to interactions with other species, even when/if multiple stressors combine additively for the organism or population alone (figure 1*d*). While the mechanisms underlying community responses to OA and multiple stressors can be ecological in nature, these effects primarily stem from physiological changes of the constituent species.

Intra- or interspecies differences in responses to multiple stressors can also lead to non-additive outcomes in ecosystem function depending on whether species' tolerance or adaptive ability covaries [16]. If functional redundancy is low or many functionally similar species have similar responses to OA, then the effects on the ecosystem may be much greater than expected based on population-level responses of single species. Exposure to multiple stressors could increase the probability of non-additive changes in ecosystem function even among communities with high variability in tolerance or adaptive ability among species to single drivers, due to the probability that more species within the community are likely to be affected as the number of stressors increases.

## 6. Moving forward

At the physiological level, a better understanding of the functional responses to single stressors, such as OA, is critical for building the theoretical framework necessary to forecast the combined effects of multiple stressors. Energy allocation concepts, commonly used to describe responses to temperatures [24], will be useful to assess the costs of physiological and adaptive responses and can provide important guidance for OA [25]. These theoretical frameworks can then inform population-level studies regarding the combined effects of multiple stressors on energy budgets and vital rates governing population dynamics. At the community level, long-term manipulative field experiments that incorporate natural variation in other environmental factors through time, as well as organismal acclimation or adaptation, may provide unparalleled insight into the emergent effects of ocean change. Long-term experiments in terrestrial grasslands can provide important guidance here [26]. Moreover, coordinated manipulative OA experiments that span a range of environmental conditions will be critical for understanding generalities in ecosystem responses [27]. In marine ecosystems, natural analogue systems, such as CO_2_ vents, could be especially useful to cross with other stressors. In all of these approaches, a focus on organismal traits or ecosystem responses that allow comparisons across levels of biological organization can reduce the likelihood of ecological surprises and improve the practical application of global change biology to conservation and management.
